# The Impact of CB2 Receptor Ligands on the MK-801-Induced Hyperactivity in Mice

**DOI:** 10.1007/s12640-017-9702-4

**Published:** 2017-01-30

**Authors:** Marta Kruk-Slomka, Izabela Banaszkiewicz, Grazyna Biala

**Affiliations:** Department of Pharmacology and Pharmacodynamics, Medical University of Lublin, Chodzki 4a Street, 20-093 Lublin, Poland

**Keywords:** Schizophrenia, Cannabis use, Endocannabinoid system, CB2 receptor ligands, MK-801, Mice

## Abstract

It has been known that there is a relationship between cannabis use and schizophrenia-related symptoms; however, it can be a subject of controversy. The involvement of CB1 receptor ligands in the schizophrenia has already been revealed and confirmed. However, there is still lack of information concerning the role of CB2 receptors in the psychosis-like effects in mice and the further studies are needed.

The aim of the present research was to study the role of the CB2 receptor ligands in the symptoms typical for schizophrenia. We provoked hyperlocomotion in mice which is analogous to positive psychosis-like effects in humans, by an acute administration of a NMDA receptor antagonist, MK-801 (0.3 and 0.6 mg/kg), a pharmacological model of schizophrenia. An acute administration of MK-801 induced the increase in locomotor activity (hyperactivity) in rodents, measured in actimeters.

We revealed that an acute injection of CB2 receptor agonist JWH 133 at the dose range (0.05–1.0 mg/kg) and CB2 receptor antagonist, AM 630 at the dose range (0.1–1.0 mg/kg) decreased locomotion of mice. An acute injection of JWH 133 (2.0 mg/kg) and AM 630 (2.0 mg/kg) had no statistical significant influence on the locomotor activity of mice. However, an acute injection of both CB2 receptor ligands (agonist and antagonist), JWH 133, at the non-effective dose of 2.0 mg/kg and AM 630 at the non-effective dose of 2.0 mg/kg, potentiated the MK-801-induced hyperactivity.

The present findings have confirmed that endocannabinoid system, not only via CB1, but also via CB2 receptors, may be involved in the schizophrenia-like responses, including hyperlocomotion in mice.

## Introduction

Schizophrenia is a chronic mental disorder that combines a variety of clinical symptoms, including positive (e.g., hallucinations, psychosis), negative (e.g., amotivation, anhedonia), and cognitive (e.g., deficits in attention and memory) symptoms (Lewis and Lieberman, 2000). As yet, the etiology of schizophrenia remains unclear. Experts support that schizophrenia is caused by several factors, including gene- and environment-related factors, as well as by an imbalance in the function of many neurotransmitters systems, e.g., dopaminergic, glutamatergic, gamma-aminobutyric(GABA)-related system, endocannabinoid system, and possibly others (Broome et al. 2005; Carlsson et al. 2004).

The endocannabinoid system, through cannabinoid (CB) receptors, and its interactions with a multitude of neurotransmitters and receptors, is involved in many physiological and physical functions, which correspond with distribution of CB receptors (Grotenhermen 2004). Currently, two types of CB receptors are known: CB1 and CB2. CB1 receptors are widely distributed in the central nervous system (CNS), especially in the limbic system and on the brain areas related to emotional responses, including basal ganglia, amygdala, cerebellum, hippocampus, and prefrontal cortex. In turn, CB2 receptors are mostly located in cells of the immune system and can be also found on the brain areas, such as cerebellum and hippocampus and in microglia (Svízenská et al. 2008). Due to localization of CB (CB1 as well CB2) receptors, endocannabinoid system is able to modulate many cognitive- and emotional-related responses in the CNS, e.g., stress, anxiety, mood, and aggressive behavior. Thus, this system plays an important role in the pathology of many CNS-related disorders, such as depression, memory loss, and schizophrenia (Grotenhermen 2004).

Recently, many literature data support that changes in the endocannabinoid system in the brain may be involved in the pathology of schizophrenia, and this system is impaired in schizophrenia. Furthermore, there is emerging evidence to support a number of associations between cannabis and psychosis. Several lines of experimental and clinical evidence point to a close relationship between endocannabinoid system and schizophrenia as cannabis use may precipitate or exacerbate the symptoms of this disease. Some studies have indicated that exposure to cannabis is associated with cognitive impairment and increased risk of developing psychosis (Arseneault et al. 2004; Moore et al. 2007). Additionally, there is evidence that the brains of people with psychosis who previously used cannabis differ significantly from those of healthy individuals (Rapp et al. 2012). Moreover, it has been revealed that people with psychosis have higher rates of cannabis use, and that there exists an association between cannabis use and schizophrenia, and other research has consistently found that cannabis use is associated with an earlier age at onset of schizophrenia (Large et al. 2011).

This relationship has been confirmed in behavioral experiments (Liu et al. 2009; Marsicano et al. 2003). A variety of animal studies found a dysregulation of endocannabinoid signaling in psychosis. For example, in animal models, it has been demonstrated that CB1 receptor agonists often induced cognitive impairments in rodents (Ferrari et al. 1999; Kruk-Slomka and Biala 2016; Pamplona and Takahashi 2006) and induced psychosis-like symptoms (Levin et al. 2012; Roser and Haussleiter, 2012). However, it is still not clear whether and why the use of cannabis causes or exacerbates psychosis.

On the other hand, due to their properties, cannabinoids appear to be a promising therapeutic target in the treatment of many diseases, such as psychosis-like symptoms. It has been revealed that antagonism of CB1 receptors generally enhanced rodent performance in variety memory tasks (Kruk-Slomka and Biala 2016; Kruk-Slomka et al. 2016a; Kruk-Slomka et al. 2016b; Lichtman 2000; Takahashi et al. 2005; Terranova et al. 1996), as well as had antipsychotic properties evaluated in animal models of schizophrenia (Almeida et al. 2014; Kruk-Slomka et al. 2016b; Levin et al. 2012; Roser and Haussleiter 2012; Radhakrishnan et al. 2014).

Since then, a number of studies have investigated the association between cannabis and psychosis. Although, the involvement of CB1 receptor ligands in the schizophrenia has already been revealed and confirmed (Kruk-Slomka et al. 2016b), there is still lack of clear evidence regarding the central mechanisms of action and effects of CB2 receptor ligands, especially the role of CB2 receptors in the processes connected with the psychosis-like symptoms. There is only a few literature data indicating a possible role of CB2 receptors in schizophrenia-related responses (Ishiguro et al. 2010; Khella et al. 2014; Ortega-Alvaro et al. 2011). Therefore, continuing in the line of our earlier studies (Kruk-Slomka et al. 2016b), in which we revealed and confirmed that CB1 receptor antagonist is able to attenuate the MK-801-induced hyperlocomotion, the aim of presented studies was to determine the potential antipsychotic status of CB2 receptor ligands and their influence on the hyperactivity in mice in this pharmacological model. We used CB2 receptor agonist, JWH 133 and CB2 receptor antagonist, AM 630. Alike the previous experiments (Kruk-Slomka et al. 2016b), we induced an increased locomotion in mice by an acute administration of *N*-methyl-d-aspartate (NMDA) receptor antagonist, MK-801, which is often used to predict the effect of many compounds with potential antipsychotic properties (Kovacic and Somanathan 2010).

The results obtained from these experiments will help to confirm the role for CB2 receptor subtype in the modulation of behaviors associated with an animal model of hyperlocomotion and schizophrenia. Following that, our results can also help to increase knowledge concerning the relationship between cannabis use and psychosis, focusing on the CB2 receptors, and to explain precise nature of these associations more clearly.

## Materials and Methods

### Animals

Male Swiss mice (Farm of Laboratory Animals, Warszawa, Poland) weighting 20–30 g were housed under laboratory conditions of controlled temperature (21 ± 1 °C), lighting (12/12 h light/dark cycle) with food (Agropol, Motycz, Poland) and tap water available ad libitum. Animals were habituated to housing conditions for 1 week and behavioral testing was carried out during the light cycle (between 8:00 and 15:00).

Mice were used only once and were drug-naive before each experiment. Experiments were performed in accordance with the National Institute of Health Guidelines for the Care and Use of Laboratory Animals and the European Community Council Directive for the Care and Use of laboratory animals of 22 September 2010 (2010/63/EU), and approved by the local ethics committee.

### Drugs

The following drugs were used:

1. *JWH 133 (0.05, 0.1, 0.25, 0.5, 1.0, 2.0 mg/kg) (Tocris, USA)—CB2 receptor agonist*
2. *AM 630 (0.1, 0.25, 0.5, 1.0, 2.0 mg/kg) (Tocris, USA)—CB2 receptor antagonist*
3. *MK-801 (0.3, 0.6 mg/kg) (Tocris, USA)—NMDA receptor antagonist*

CB2 receptor ligands and MK-801 were suspended in the 1% solution of polyoxyethylenesorbitan monooleate (Tween 80) (Sigma, St. Louis, MO, USA) and then diluted in a 0.9% saline solution (NaCl). All solutions were injected intraperitoneally (ip) at a volume of 10 ml/kg body weight. Saline plus Tween 80 was used as a control solution (vehicle), at the same volume and by the same route of administration. Fresh drug solutions were prepared on each day of experimentation.

Experimental doses of drugs used and procedures were selected on the basis of literature data (Bubenikova-Valesova et al. 2010; Kovacic and Somanathan 2010; Nestler and Hyman 2010; Mohn et al. 1999; Zhou et al. 2012), our previous experiments (Kruk-Slomka et al. 2015; Kruk-Slomka and Biala 2016; Kruk-Slomka et al. 2016a; Kruk-Slomka et al. 2016b), and preliminary studies. The doses of MK-801 as well as the scheme of treatment was based on our previous experiments (Kruk-Slomka et al. 2016b).

### Experimental Procedures

Schizophrenia-like behavior was assessed using the commonly accepted pharmacological animal model of schizophrenia based on psychotic properties of MK-801***.*** Mice were acutely injected with MK-801 inducing hyperlocomotion which correlates with the psychomotor agitation seen in schizophrenia patients (Bubenikova-Valesova et al. 2010; Nestler and Hyman 2010).

Firstly, the influence of CB2 receptor ligands, JWH 133 and AM 630, on the locomotor activity of mice was investigated and then the impact of JWH 133 and AM 630 on the hyperactivity provoked by MK-801 was estimated. Locomotion of mice was measured in actimeters.

#### Locomotion

Locomotion of mice was recorded individually in round actometer cages (Multiserv, Lublin, Poland; 32 cm in diameter, two light beams) kept in a sound-attenuated experimental room. Two photocell beams, located across the axis, automatically measured animal’s movements. The horizontal locomotor activity, i.e., the number of photocell beam breaks, was automatically measured with a 20-min interval for 200 min (Mohn et al. 1999; Zhou et al. 2012).

### Treatment

#### For Psychotic-like Symptoms

Horizontal locomotor activity was measured immediately after an acute injection of JWH 133 (0.05, 0.1, 0.25, 0.5, 1.0, 2.0 mg/kg, ip), AM 630 (0.1, 0.25, 0.5, 1.0, 2.0 mg/kg, ip), or vehicle for the control group. Next, we evaluated the impact of an acute administration of JWH 133 (2.0 mg/kg, ip) or AM 630 (2.0 mg/kg, ip) on the hyperlocomotion of mice provoked by an acute MK-801 (0.3 and 0.6 mg/kg, ip). For this purpose, JWH 133, AM 630, or vehicle were administered 15 min before injection of MK-801 or vehicle. The mice were then tested immediately after the last injection.

### Statistical Analysis

The statistical analysis were performed using two-way ANOVA—for the factors of time, drug treatment (JWH 133, AM 630, and/or MK-801), and time/drug treatment interactions for the locomotor effects.

Post hoc comparison of means was carried out with the Bonferroni’s test for multiple comparisons, when appropriate. The data were considered statistically significant at confidence limit of *p* < 0.05. ANOVA analysis with Bonferroni’s post tests were performed using GraphPad Prism version 5.00 for Windows, GraphPad Software, San Diego California USA, www.graphpad.com.

For the psychotic-like symptoms, the horizontal locomotor activity, i.e., the number of photocell beam breaks, was measured.

## Results

First, we evaluated the influence of CB2 receptor ligands (agonist and antagonist) on the locomotion of mice in actimeters. An acute injection of JWH 133, CB2 receptor agonist decreased locomotion of mice at the dose range 0.05–1.0 mg/kg. Similarly, an acute injection of AM 630. CB2 receptor antagonist, decreased locomotion of mice at the dose range 0.1–1.0 mg/kg. In turn, an acute injection of both JWH 133 at the dose of 2.0 and AM 630 at the dose of 2.0 had no influence on the locomotion of mice. Therefore, these non-effective doses of JWH 133 and AM 630 (2.0 mg/kg) have been chosen to the next experiments dealing MK-801.

### The Influence of CB2 Receptor Agonist, JWH 133, on the Locomotor Activity of Mice

Two-way ANOVA analyses revealed that there was statistically significant effect caused by time [*F*(10,418) = 44.04, *p* < 0.0001] and JWH 133 treatment [*F*(6418) = 16.30, *p* < 0.0001], but there was no statistically significant effect caused by interactions between time and JWH 133 treatment [*F*(60,418) = 0.94, *p* = 0.6122]. The Bonferroni’s test revealed that an acute injection of JWH 133, at the dose range (0.05–1.0 mg/kg), significantly decreased locomotion in mice in comparison to the vehicle-treated control group between the following minutes of the experiment as follows:

1. Between 140 and 200 min of experiment for the dose of 0.05 mg/kg of JWH 133: 140 min (*p* < 0.05), 160 min (*p* < 0.01), and 180–200 min (*p* < 0.001)
2. Between 100 and 200 min of experiment for the dose of 0.1 mg/kg of JWH 133: 100 min (*p* < 0.05), 120 min (*p* < 0.01), and 140–200 min (*p* < 0.001)
3. Between 180 and 200 min of experiment for the dose of 0.25 mg/kg of JWH 133: *p* < 0.01
4. Between 180 and 200 min of experiment for the dose of 0.5 mg/kg of JWH 133: 180 min (*p* < 0.05), 200 min (*p* < 0.01)
5. Between 120 and 200 min of experiment for the dose of 1.0 mg/kg of JWH 133: 120 min (*p* < 0.05), 140–180 min (*p* < 0.01), 200 min (*p* < 0.001)

**Fig. 1:**
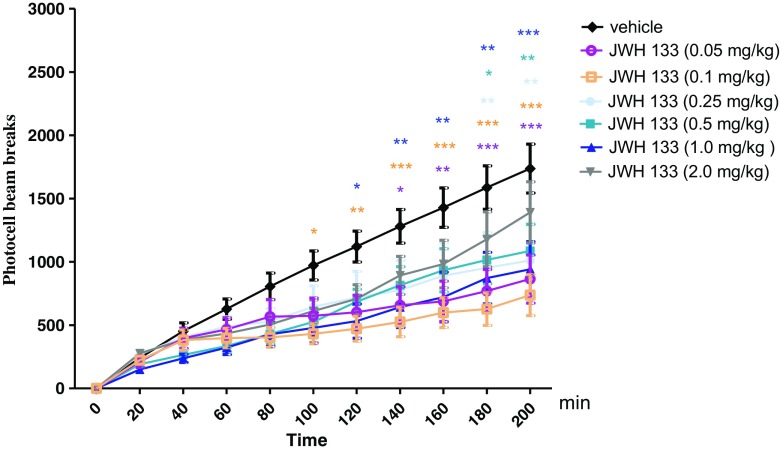
Effects of an acute JWH 133 or vehicle administration on the locomotor activity of mice. JWH 133 (0.05, 0.1, 0.25, 0.5, 1.0, and 2.0 mg/kg; ip) or vehicle were injected immediately before the test; *n* = 8–12; the means ± SEM; *p < 0.05; **p < 0.01; ***p < 0.001 vs. vehicle-treated control group; Bonferroni’s test

JWH 133 at the dose of 2.0 mg/kg had no influence on the locomotor activity of mice in comparison to the vehicle-treated control group (Fig. 1).

### The Influence of CB2 Receptor Antagonist, AM 630 on the Locomotor Activity of Mice

Two-way ANOVA analyses revealed that there was statistically significant effect caused by time [*F*(10,341) = 40.07, *p* < 0.0001] and AM 630 treatment [*F*(5341) = 20.01, *p* < 0.0001], but there was no statistically significant effect caused by interactions between time and AM 630 treatment [*F*(50,341) = 1.20, *p* = 0.1827]. The Bonferroni’s test revealed that an acute injection of AM 630 at the dose range (0.1–1.0 mg/kg) significantly decreased locomotion in mice in comparison to the vehicle-treated control group between the following minutes of the experiment as follows:

1. Between 140 and 200 min of experiment for the dose of 0.1 mg/kg of AM 630: 140–160 min (*p* < 0.05), 180 min (*p* < 0.01), and 200 min (*p* < 0.001)
2. Between 100 and 200 min of experiment for the dose of 0.25 mg/kg of AM 630: 100 min (*p* < 0.05), 120 min (*p* < 0.01), and 140–200 min (*p* < 0.001)
3. Between 120 and 200 min of experiment for the dose of 0.5 mg/kg of AM 630: 120 min (*p* < 0.05), 140 min (*p* < 0.01), and 160–200 min (*p* < 0.001)
4. Between 160 and 200 min of experiment for the dose of 1.0 mg/kg of AM 630: 160 min (*p* < 0.05) and 180–200 min (*p* < 0.01)

**Fig. 2:**
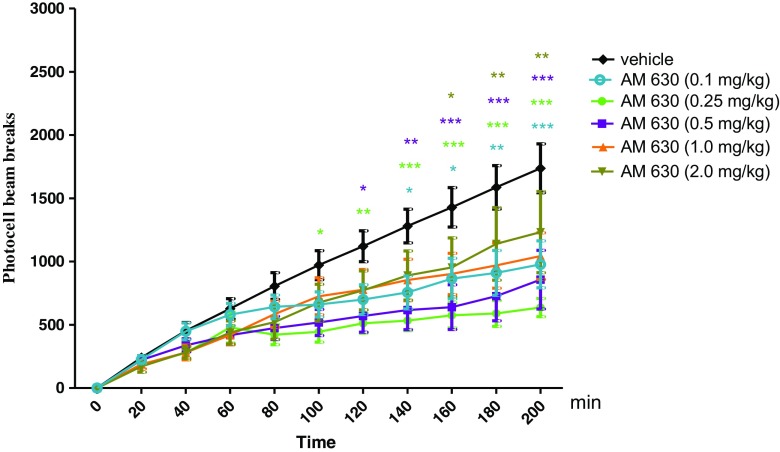
Effects of an acute AM 630 or vehicle administration on the locomotor activity of mice. AM 630 (0.1, 0.25, 0.5, 1.0, and 2.0 mg/kg; ip) or vehicle were injected immediately before the test; *n* = 8–12; the means ± SEM; *p < 0.05; **p < 0.01; ***p < 0.001 vs. vehicle-treated control group; Bonferroni’s test

AM 630 at the dose of 2.0 mg/kg had no influence on the locomotor activity of mice in comparison to the vehicle-treated control group (Fig. 2).

Then, we induced the hyperlocomotion which mimics positive symptom characteristic for schizophrenia, by an acute administration of MK-801, and evaluated the influence of CB2 receptor ligands on this MK-801-related hyperactivity. Based on our previously conducted experiments (Kruk-Slomka et al. 2016b) as well as on the results obtained from the experiments described above, the effective doses of MK-801 (0.3 and 0.6 mg/kg) and non-effective dose of JWH 133 (2.0 mg/kg) and AM 630 (2.0 mg/kg) were then chosen for the next behavioral experiment evaluating the involvement of CB2 receptors on MK-801-induced hyperactivity.

In these experiments, we revealed that an acute injection of both CB2 receptor agonist and antagonist had influence on the MK-801-induced hyperlocomotion of mice. The non-effective dose of JWH 133 (2.0 mg/kg) potentiated the hyperlocomotion of mice provoked by an acute injection of MK-801 at the dose of 0.6 mg/kg. Similarly, an acute injection of non-effective dose of AM 630 (2.0 mg/kg) potentiated the hyperlocomotion of mice provoked by an acute injection of MK-801 both at the dose of 0.3 and 0.6 mg/kg, as described below.

### The Influence of the Administration of JWH 133 on the Hyperactivity of Mice Provoked by an Acute Administration of MK-801

**Fig. 3:**
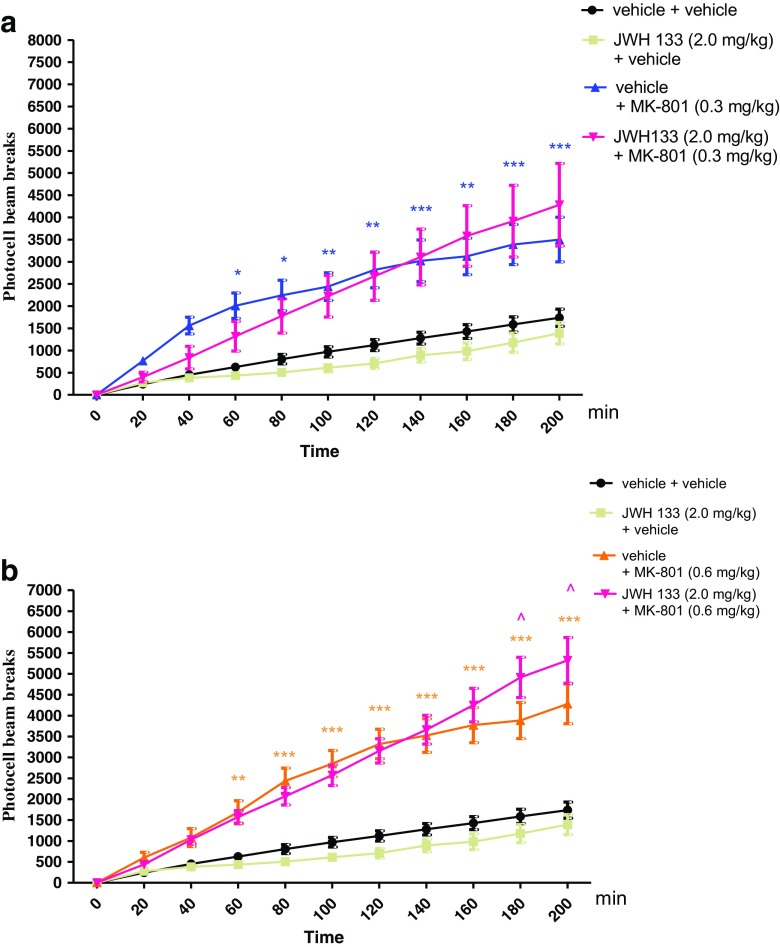
Effect of JWH 133 on MK-801-induced hyperactivity in mice. Non-effective dose of JWH 133 (2.0 mg/kg, ip) or vehicle were administered 15 min prior to vehicle or effective (0.3 mg/kg, ip) (**a**) and (0.6 mg/kg, ip) (**b**) dose of MK-801. After the last injection, the mice were then tested in actimeters; *n* = 8–12; the means ± SEM; *p < 0.05; **p < 0.01; ***p < 0.001 vs. vehicle/vehicle-treated group; ^*p* < 0.05 vs. vehicle/MK-801(0.6 mg/kg)-treated group Bonferroni’s test

Two-way ANOVA analyses revealed that there was statistically significant effect caused by time [*F*(10,264) = 32.84, *p* < 0.0001] and drug (MK-801 (0.3 mg/kg) and/or JWH 133 (2.0 mg/kg) treatment [*F*(3264) = 81.27, *p* < 0.0001], as well as caused by interactions between time and drug treatment [*F*(30,264) = 2.73, *p* < 0.0001]. The post hoc Bonferroni’s test confirmed that an acute injection of MK-801 at the dose of 0.3 mg/kg significantly increased locomotor activity of mice between 60 and 200 min of experiment as compared with the vehicle/vehicle-injected control group (for 60–80 min of experiments *p* < 0.05, for 100–120 min *p* < 0.01, for 140 min *p* < 0.001, for 160 min *p* < 0.01, and for 180–200 min *p* < 0.001). JWH 133 (2.0 mg/kg) had no influence on MK-801 (0.3 mg/kg)-induced hyperactivity (Fig. 3a).

For the second dose of MK-801 used (0.6 mg/kg), two-way ANOVA analyses revealed that there was statistically significant effect caused by time [*F*(10,264) = 80.87, *p* < 0.0001], and drug (MK-801 (0.6 mg/kg) and/or JWH 133 (2.0 mg/kg) treatment [*F*(3264) = 213.39, *p* < 0.0001], as well as by interactions between time and drug treatment [*F*(30,264) = 8.72, *p* < 0.0001]. The post hoc Bonferroni’s test revealed that MK-801 at the dose of 0.6 mg/kg significantly increased locomotor activity of mice in actimeters between 60 and 200 min of experiment (for 60 min of experiment *p* < 0.01, for 80–200 min *p* < 0.001), in comparison to the vehicle/vehicle-treated mice. Moreover, this hyperactivity provoked by MK-801 (0.6 mg/kg) was potentiated by JWH 133 (2.0 mg/kg) between 180 and 200 min of experiment (*p* < 0.05 vs. vehicle/MK-801 (0.6 mg/kg)-treated mice) (Fig. 3b).

### The Influence of the Administration of AM 630 on the Hyperactivity of Mice Provoked by an Acute Administration of MK-801

**Fig. 4:**
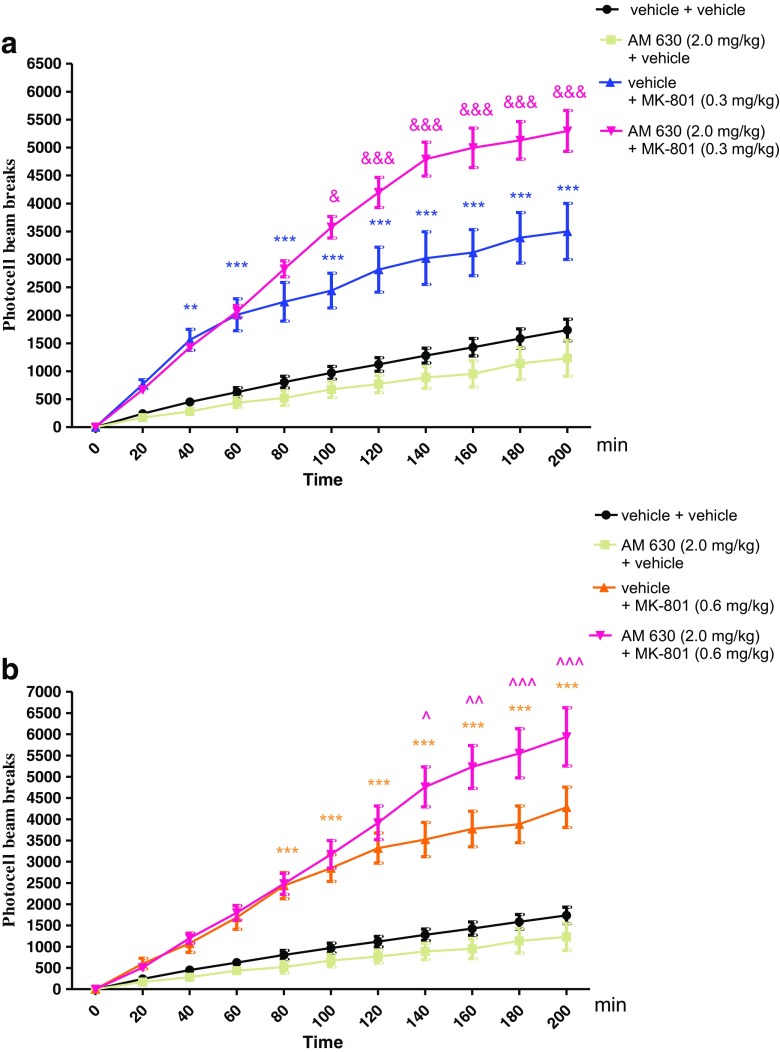
Effect of AM 630 on MK-801-induced hyperactivity in mice. Non-effective dose of AM 630 (2.0 mg/kg, ip) or vehicle were administered 15 min prior to vehicle or effective (0.3 mg/kg, ip) (**a**) and (0.6 mg/kg; ip) (**b**) dose of MK-801. After the last injection, the mice were then tested in actimeters; *n* = 8–12; the means ± SEM; **p < 0.01; ***p < 0.001 vs. vehicle/vehicle-treated group; &*p* < 0.05; &&&*p* < 0.001 vs. vehicle/MK-801(0.3 mg/kg)-treated group; ^*p* < 0.05; ^^*p* < 0.01; ^^^*p* < 0.001 vs. vehicle/MK-801(0.6 mg/kg)-treated group; Bonferroni’s test

Two-way ANOVA analyses revealed that there was statistically significant effect caused by time [*F*(10,242) = 66.29, *p* < 0.0001] and drug (MK-801 (0.3 mg/kg) and/or AM 630 (2.0 mg/kg) treatment [*F*(3242) = 261.74, *p* < 0.0001], as well as caused by interactions between time and drug treatment [*F*(30,242) = 8.11, *p* < 0.0001]. The post hoc Bonferroni’s test confirmed that an acute injection of MK-801 at the dose of 0.3 mg/kg significantly increased locomotor activity of mice between 40 and 200 min of experiment as compared with the vehicle/vehicle-injected control group (for 40 min of experiment *p* < 0.01, for 60–200 min *p* < 0.001). Moreover, this hyperactivity provoked by MK-801 (0.3 mg/kg) was potentiated by an acute injection of AM 630 (2.0 mg/kg) between 100 and 200 min of experiment (for 100 min of experiment *p* < 0.05, for 120–200 min of experiment *p* < 0.001 vs. vehicle/MK-801 (0.3 mg/kg)-treated mice) (Fig. 4a).

Similarly, for the second dose of MK-801 used (0.6 mg/kg), two-way ANOVA analyses revealed that there was statistically significant effect caused by time [*F*(10,242) = 67.19, *p* < 0.0001] and drug (MK-801 (0.6 mg/kg) and/or AM 630 (2.0 mg/kg) treatment [*F*(3242) = 213.41, *p* < 0.0001], as well as by interactions between time and drug treatment [*F*(30,242) = 8.58, *p* < 0.0001. The post hoc Bonferroni’s test revealed that MK-801 at the dose of 0.6 mg/kg significantly increased locomotor activity of mice in actimeters between 80 and 200 min of experiment (*p* < 0.001), in comparison to the vehicle/vehicle-treated mice. Moreover, this hyperactivity provoked by MK-801 (0.6 mg/kg) was increased by AM 630 (2.0 mg/kg) between 140 and 200 min of experiment (for 140 min of experiment *p* < 0.05, for 160 min *p* < 0.01, for 180–200 min of experiment *p* < 0.001 vs. vehicle/MK-801 (0.6 mg/kg)-treated mice) (Fig. 4b).

## Discussion

The aim of the present research was to determine the involvement of the endocannabinoid system, through CB2 receptors, in the hyperlocomotion of mice, provoked by an acute injection of NMDA receptor antagonist, MK-801, as an animal pharmacological model of schizophrenia.

We revealed that an acute injection of AM 630, CB2 receptor antagonist, used in our experiments at the doses 0.1–1.0 mg/kg, but not at the dose of 2.0 mg/kg, decreased locomotion of mice. An acute injection of AM 630 (2.0 mg/kg) of its own had no statistical significant influence on the locomotor activity of mice. However, in the next step, we revealed that an acute injection of AM 630, at the non-effective dose of 2.0 mg/kg, potentiated MK-801-induced hyperactivity. What is of interest, we revealed that an acute injection of JWH 133, CB2 receptor agonist, used in our experiments at the doses 0.05–1.0 mg/kg, but not at the dose of 2.0 mg/kg, also decreased locomotion of mice. An acute injection of JWH 133 (2.0 mg/kg) of its own had no statistical significant influence on the locomotor activity of mice. However, an acute injection of JWH 133 at the non-effective dose of 2.0 mg/kg intensified the MK-801-provoked hyperactivity.

The first CB2 receptor ligand-related effect that should be discussed is associated with the fact that both the CB2 agonist (2.0 mg/kg; not a lower dose) and CB2 antagonist (2.0 mg/kg; not a lower dose) altered MK-801 locomotor effects (similar effects). The second interest CB2 receptor ligand effect should focused on the fact that these cannabinoid compounds had no effect of their own (i.e., without MK-801 injection) on locomotor activity.

The increase of MK-801 induced hyperactivity caused by both CB2 antagonist and CB2 agonist obtained in our studies seems to be very important. The same direction of action we have is revealed in our previous studies. We have shown the enhancement of memory and learning processes by an acute administration of CB2 receptor agonist (JWH 133 at the doses of 0.5; 1.0 and 2.0 mg/kg) and antagonist (AM 630 at the dose of 1.0; 2.0 and 3.0 mg/kg) assessed in the passive avoidance (PA) test (Kruk-Slomka et al. 2016a). Similarly, we have revealed that an acute administration of both JWH 133 (0.5 and 1.0 mg/kg) and AM 630 (0.5 mg/kg) exhibited antidepressant action in the forced swimming test (FST) (Kruk-Slomka et al. 2015). These results confirmed in our present and previous cited experiments may be connected with pharmacokinetic properties of used CB2 receptor ligands, especially the CB2-selective agent, AM 630. The pharmacological properties of AM 630 are complex. It has been shown that AM 630 behaves as inverse agonist rather than as “silent” antagonist. The inverse efficacy at CB2 receptors but also the CB2/CB1 affinity ratio has been indicated for AM 630 (CB2/CB1 affinity = 165). Thus, AM 630 has been found to behave as an inverse agonist at CB2 receptors as well as an inverse agonist at CB1 receptors (Bolognini et al. 2012; Ross et al. 1999).

Naturally, the administration of different doses of selective CB2 receptor agonists/antagonists provides some information for a neurophysiological role of CB2 receptors in the brain, although non-selective effects at these doses cannot be ruled out. What is of interest, it has been revealed that CB2 receptor antagonist, SR144528, causes biphasic effects on the locomotion of mice, increasing spontaneous locomotor activity in the DBA/2 mouse at low doses (1.0–10.0 mg/kg) and decreasing this activity at high doses (20.0 mg/kg) (Onaivi et al. 2008). Although, Sain et al. (2009) as well as Whiteside et al. (2005) revealed that genetic deletion of the CB2 receptor has not been connected with any change in motor effects. On the other hand, increasing doses of the CB2 receptor agonist, JWH-015, decreased locomotor activity and stereotyped behavior what was depended on the gender of animals (Onaivi et al. 2006). Similarly, typical hypolocomotion was observed after administration of an alternative CB2 receptor agonist GW 405833 (Valenzano et al. 2005) but the injection of the selective CB2 receptor agonists HU 308 and AM 1214, at doses provoking significant antinociceptive effects, did not affect locomotor activity (Hanus et al. 1999; Malan et al. 2001).

Additionally, Xi et al. (2011) have revealed that intranasal or intra-accumbens local administration of CB2 receptor agonist, JWH 133, dose-dependently inhibits cocaine-enhanced locomotion in wild-type (WT) and CB1 receptor-knockout (CB1−/−), but not CB2−/− mice. This inhibition is mimicked by GW 405833, another CB2 receptor agonist with a different chemical structure, and is blocked by AM 630, a selective CB2 receptor antagonist. Intra-accumbens administration of JWH 133 alone dose-dependently decreases, while intra-accumbens AM 630 elevates locomotion in WT and CB1−/− mice, but not in CB2−/− mice.

Our results indicating that CB2 receptor ligands did not affect locomotor activity in the dose used (2.0 mg/kg) are consistent also with other literature data (Ishiguro et al. 2010; Kim and Li 2015; Khella et al. 2014; Onaivi 2009; Ortega-Alvaro et al. 2011). The data indicating the role of CB2 receptor agonist in the MK-801-induced disruptions of PPI in mice seem to be interesting (Khella et al. 2014). In this cited experiment, JWH 015, a CB2 receptor agonist had no significant effect on prepulse inhibition (PPI) alone but reversed disruptions in PPI induced by MK-801. This effect was attenuated by the injection of AM 630, but not by AM 251, CB1 receptor antagonist, suggesting an involvement of CB2 receptors in these effects. Additionally, JWH 203, the mixed CB1/CB2 receptor agonist only partially reduced MK-801-induced PPI disruptions but neither AM 630 nor AM 251 had any significant effect alone or on MK-801-induced disruptions in PPI (Khella et al. 2014). In turn, Ishiguro et al. (2010) have confirmed that AM 630 exacerbated MK-801- or methamphetamine-induced disturbance of PPI and hyperactivity in C57BL/6JJmsSlc mice. Thus, we can suspect that the inhibition of the activity of CB2 receptor by AM 630 at the dose of 2.0 mg/kg is not able to contribute to the emergence of symptoms of schizophrenia. However, the inhibition of the function of receptor CB2 in combination with other risk factors (e.g., the impairment of glutamatergic transmission by the use of NMDA receptor antagonist—MK-801) may provoke or potentiate the symptoms of schizophrenia, perhaps in the people predisposed to schizophrenia. Perhaps, the possible explanation of our results is connected with the fact that pharmacological ligands targeting CB2 receptor (JWH 133 and AM 630) in the normal conditions (without injection of MK-801) and in the highest dose used (2.0 mg/kg) had no influence on the locomotor activity in mice. But in the presence of some risk factor of schizophrenia in animals (e.g., administration of MK-801), CB2 expression in the brain is changed and CB2 receptor ligands exhibit potent antipsychotic (anti-hyperactivity) effects. Naturally, we can only support these conclusions and more detailed knowledge of these effects needs further investigations.

The mechanism of action of CB2 receptor ligands in psychosis-like responses in mice is still unclear. Although, a number of studies have investigated the association between cannabis and psychosis, many questions remain unanswered. It has been known that endocannabinoid system, mainly via CB1 receptors, is involved in pathomechanisms of schizophrenia (Barzegar et al. 2015; Kruk-Slomka et al. 2016b; Kucerova et al. 2014; Levin et al. 2012; Roser and Haussleiter 2012), while the CB2 receptors locating outside the CNS are involved in the processes related to the function of the immune system (Fukuda et al. 2014; Pertwee 2010; Wright et al. 2008). However, recent studies have provided evidence that CB2 receptors may also be arranged outside the cells of the immune system. Literature data suggested that these receptors can also be found in various brain regions of humans and many animal species (Benito et al. 2008; Zhang et al. 2014). The studies on the biochemical and histological level have revealed that the CB2 receptors are located in the neuronal progenitor cells, neurons, glial chambers, and endothelium. There is also evidence for the expression of CB2 receptors in areas of the brain that are particularly pertinent to the context of schizophrenia, such as regions involved in sensorimotor gating (Onaivi 2009; Racz et al. 2008; Zhang et al. 2014), but the functional significance of this discovery has not been established yet.

Following that, the functional role of central CB2 receptors is not fully elucidated yet. Recent evidence obtained from pharmacological (Ishiguro et al. 2010; Kim and Li 2015; Onaivi 2009; Ortega-Alvaro et al. 2011) and genetic studies (Ortega-Alvaro et al. 2011) suggest that centrally expressed CB2 receptors are involved in many processes of neuropsychiatric disorders, such as behavior characteristic of schizophrenia, impulsive behavior, locomotor activity, stereotyped behaviors, anxiety-, pain-, and memory-related processes (Khella et al. 2014; Kim and Li 2015; Kruk-Slomka et al. 2016a). Moreover, literature data (Kruk-Slomka et al. 2015; Onaivi 2009) revealed the possible involvement of CB2 receptors in depression, which may be of importance in many psychiatric disorders connecting with emotional imbalance, including schizophrenia. What is more, the literature data indicated that the deletion of the gene encoding CB2 receptors may lead to neurochemical changes which can be a consequence of a behavioral disorder mentioned above (Ishiguro et al. 2010; Ortega-Alvaro et al. 2011).

We confirmed in results presented in this paper that both stimulation and blockade of CB2 receptors is able to modulate locomotor activity of mice that correlates with the neuropsychiatric effects. It has been known that the neuropsychiatric effects of endocannbinoid system, via CB1 and CB2 receptors, are associated with the modulation of different neurotransmitter systems, such as dopaminergic system, glutamatergic system, or GABA-related system (Broome et al. 2005; Carlsson et al. 2004). A variety of pre-clinical and clinical studies have indicated that mainly CB1 receptors participate in many central pathways connected with psychosis-like state through influence on the glutamatergic transmission (Barzegar et al. 2015; Kruk-Slomka et al. 2016b). However, in the case of CB2 receptors, many literature data suggested an important role of CB2 receptors, locating in the specific brain areas, mainly on the dopaminergic neurons in the ventral tegmental area (VTA). Electrophysiological studies have demonstrated that CB2 receptor activation by JWH 133 or other CB2 receptor agonists leads to inhibition of dopaminergic neuron activation in the VTA. Therefore, these CB receptors play an important role in the modulation of dopaminergic system and are involved in the behaviors connecting with dopaminergic system-related disorders, such as schizophrenia, anxiety, depression, and Parkinson disease (Zhang et al. 2014). Additionally, the endocannabinoid system via CB2 receptors can be associated with schizophrenia-like response, due to the fact that this type of receptors is responsible for the increase of amount of annandamide, the main endocannabinoid occurring in the brain. The clinical studies confirmed that the clinical remission of schizophrenia may be connected with the significant decrease in the level of anandamide and messenger ribonucleic acid (mRNA) encoding CB2 receptors (Ishiguro et al. 2010; Vigano et al. 2008). However, there are significant species differences in CB2 receptor mRNA splicing and expression, protein sequences, and CB2 receptor ligand-related in mice and rats (Zhang et al. 2015) indicating that the cellular mechanisms underlying these actions are still unclear; therefore, the function of cannabinoid CB2 receptors in the brain have been subject to debate. Following that, the influence of CB2 agonists and antagonists on hyperlocomotion of mice is controversial and inconsistent, for this reason need further investigation.

## Conclusion

The results presented in this paper, as well as the cited literature data, allow evaluating the possible relationship between endocannbinoid system and positive symptoms of schizophrenia, focusing on the CB2 receptors. It can also be assumed that modulation of the CB2 receptor function in combination with other risk factors (e.g., disturbances in the glutamatergic transmission) may lead to the onset or intensification of the symptoms of schizophrenia. However, further behavioral studies would be required to conclusively establish the role of CB2 receptors in schizophrenia and would be essential in understanding and developing the pharmacological profile of novel antipsychotics in the future.

## Abbreviations

Δ9-THC-Δ9: Tetrahydrocannabinol
CB1: Cannabinoid receptor of type 1
CB2: Cannabinoid receptor of type 2
(CB1−/−): CB1 receptor knockout mice
(CB2−/−): CB2 receptor knockout mice
CB: Cannabinoid
CBD: Cannabidiol
CNS: Central nervous system
GABA: ɣ-Amino-butyric acid
ip: Intraperitoneally
mRNA: Messenger ribonucleic acid
NMDA: *N*-methyl-d-aspartate
PPI: Prepulse inhibition
WT: Wild type
VTA: Ventral tegmental area
